# Supplementary material for: Humoral Response Kinetics and Cross-Immunity in Hospitalized Patients with SARS-CoV-2 WT, Delta, or Omicron Infections: A Comparison between Vaccinated and Unvaccinated Cohorts
Source: Vaccines (Basel). 2023 Dec 1;11(12):1803. doi: 10.3390/vaccines11121803 (PMC10747008; doi:10.3390/vaccines11121803)
Supplement: Supplementary file 1 [file vaccines-11-01803-s001.zip › vaccines-2730738-supplementary.pdf]

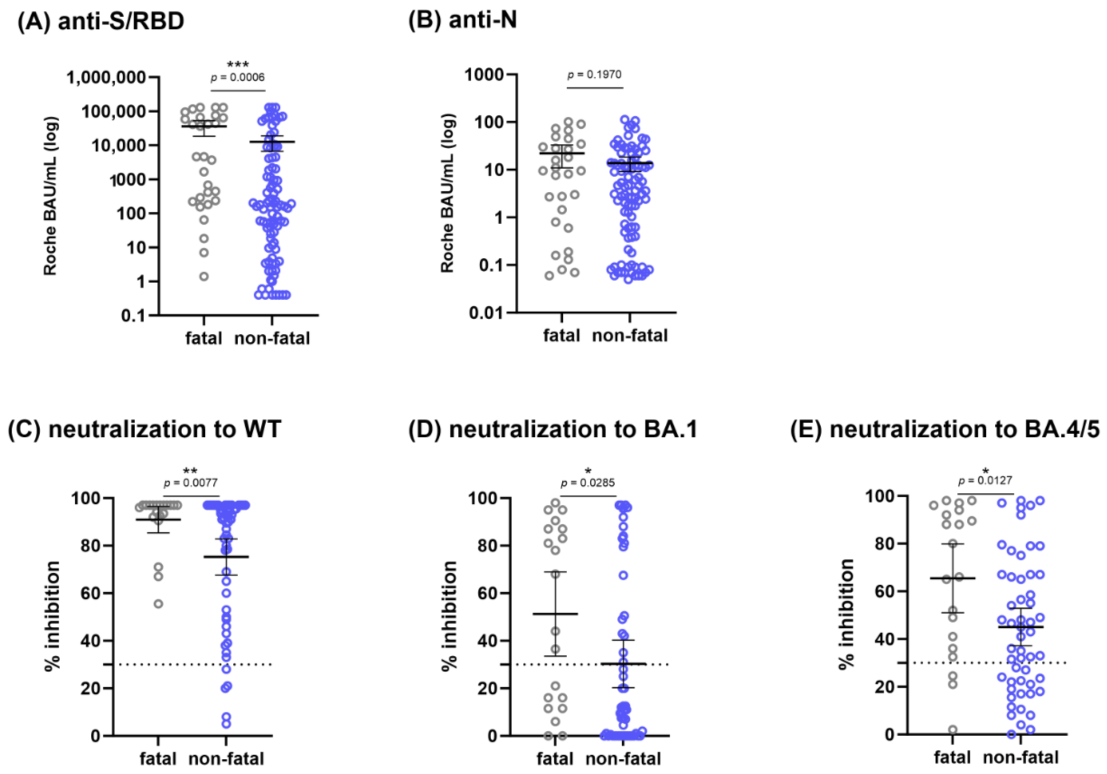

Figure S1. Comparison of humoral immune responses between fatal and non-fatal patients.
